# Supplementary material for: Optimization of 3D printing supply chain in the era of live streaming e-commerce
Source: PLoS One. 2024 May 14;19(5):e0303218. doi: 10.1371/journal.pone.0303218 (PMC11093336; doi:10.1371/journal.pone.0303218)
Supplement: Attachment — Submitted filename: Response to Reviewer.docx [file pone.0303218.s001.docx]

| **Response to Reviewer #1** | | |
| --- | --- | --- |
| **No** | **Comments** | **Response** |
| 1 | The Abstract should be improved. The more about motivation, methods, results, and findings is missing. “Live streaming e-commerce”must be mentioned in the abstract. | We would like to thank the esteemed reviewer#1 for the constructive comment. We have rewritten the abstract based on relevant feedback and added more information about motivation, methods, results, and findings. Please refer to the abstract section. |
| 2 | The last paragraph in the introduction section should be a structure of the paper (several sentences for each section). This is missing. | We indeed appreciate our esteemed reviwer#1 for his/her micro level through observation and constructive feedback provided. We have added the relevant sentences in the last paragraph in the introduction section. Please check. |
| 3 | It is necessary to understand the purpose and aim of the paper as well as its "position" in previous research (also gap analysis). | We thank our esteemed Reviewer #1 for his/her thoughtful analysis of the contribution of the paper. We have added the relevant sentences in the last paragraph in the introduction section to emphasize its position in live streaming e-commerce research . Please check. |
| 4 | The separate section Practical and Theoretical Implications (or Discussion) is missing. The existing section Discussion is very modest. This confirms the lack of scientific and practical contribution | Thank you very much for the constructive criticism. We have separated and rewritten the discussion and the conclusion section. We have added more contents in the discussion section. Please check. |
| 5 | The paper is not written in a scientific manner. For example, see Section 3. Explanations are missing. | We have added necessary level 3 headings to the third part, rearranged the order of the content, and rewritten and deleted parts that are prone to confusion, striving to provide reviewers with a more intuitive understanding of the focus of our research. Please check. |
| 6 | The methodology is not clear and sufficiently explained. Argumentation is missing. | We have provided a detailed supplement and explanation of the system dynamics methods used in section 2.1. Thank you very much for your reminder and guidance. |
| 7 | Results and findings are questionable | We have significantly supplemented the part of conclusion, please review. |
| 8 | Scientific contributions are also questionable. | We have significantly supplemented the part of conclusion, please review. |
| 9 | Also, the conclusion section is not on a satisfactory level. The conclusion in scientific papers is very important. | We hope that our modifications can allow the reviewer#1 to understand the dedication and innovative contributions we have put into this research. Please review. |
| 10 | Limitations of your research must be emphasized | We have significantly supplemented the limitations and future research, please review. |
| 11 | Future research directions must be reinforced. | We have significantly supplemented the limitations and future research, please review. |
| 12 | Suggested references | The suggested references have been added in the manuscript and cited in the proper place. |

| **Response to Reviewer #2** | | |
| --- | --- | --- |
| **No** | **Comments** | **Response** |
| 1 | The current abstract should be greatly improved as it does not reveal the novelty of the current study. | We appreciate Reviewer's insightful observation of the abstract. We have rewritten the abstract based on relevant feedback and added more information about motivation, methods, results, and findings. Please refer to the abstract section. |
| 2 | Can the authors provide more details on how the utility equilibrium point between private and corporate live streaming 3D printing was calculated? | We sincerely thank the esteemed reviewer #2 for his/her insightful comments on the key point of the manuscript. We have added a variable Z to reflect the utility equilibrium point. Hope this setting will satisfy the reviewer #2. |
| 3 | What specific criteria were considered when comparing the suitability of corporate and private live 3D printing modes for polymer and polymer-metal hybrid printing? | As the explanation in 2.3.2, the criteria is the ulitities of printing time, cost and quality. |
| 4 | Could you elaborate on the analysis of consumer needs that led to the conclusion that private live 3D printing mode can still meet the needs of some consumers? | We have rewritten the section of discussion. Please review and find the analysis in 4.2. |
| 5 | The introduction of Section 3.1 is very poor. Improve it. | We have added necessary level 3 headings to the third part, rearranged the order of the content, and rewritten and deleted parts that are prone to confusion, striving to provide reviewers with a more intuitive understanding of the focus of our research. Please check. |
| 6 | In what ways does the transition to a corporate live 3D printing mode create a new consumption model, and how is this different from the current models? | We have rewritten the section of discussion. Please review and find the analysis in 4.1. |
| 7 | How does the proposed shift in 3D printing modes contribute to making the global economic system more resilient, flexible, and localized? Can you provide specific examples or mechanisms? | We have rewritten the section of introduction. Please review and find the analysis in 1.1. |
| 8 | What are the potential implications for localized production and supply chain dynamics as a result of the shift to a corporate live 3D printing mode? | We have rewritten the section of discussion. Please review and find the analysis in 4.1.2. |
| 9 | In terms of comparison with previous studies, the discussion is not acceptable. It must be greatly improved during revision. | We have rewritten the section of discussion. Please review. We hope that our modifications can allow the reviewer#2 to catch the key point of live 3DP. |
| 10 | Can you elaborate on how the proposed changes are expected to stimulate further development in 3D printing technology? Are there specific areas or aspects that will see accelerated growth? | We have rewritten the section of conclusion. Please review the 5.2 and find our suggestions. We hope that our modifications can allow the reviewer#2 to catch the key point of live 3DP. |
| 11 | Did the study take into account the environmental impact of the different 3D printing modes, especially considering the increasing focus on sustainability in manufacturing? | We have rewritten the section of discussion. Please review the section 4.1.2 and 4.2.2. The environmental impact of the different 3DP modes have been compared. |
| 12 | What limitations or constraints were encountered during the research, and how might they have influenced the study's findings and conclusions? | We have rewritten the section of conclusion. Please review the 5.2.1 and find our findings of limitations. We hope that our modifications can allow the reviewer#2 to catch the key point of live 3DP. |
| 13 | How do the research results suggest potential changes in industry practices related to 3D printing, and are there specific recommendations for practitioners based on the findings? | We have rewritten the section of conclusion. Please review the 5.2.2 and find our findings of future works. We hope that our modifications can allow the reviewer#2 to catch the key point of live 3DP. |
